# Quarterly Report (No. 4;) Being a List of the Medical Cases, Admitted from the 10th of November, 1822, to the 10th of February, 1823, at the Westminster General Dispensary

**Published:** 1823-03

**Authors:** R. Macleod

**Affiliations:** one of the Physicians to that Institution.


### STATISTICAL MEDICINE.

ART. I.—*Quarterly Report (No. 4;) being a List of the Medical Cases, admitted from the 10th of November, 1822, to the 10th of February, 1823, at the Westminster General Dispensary.* By R. MACLEOD, M.D. one of the Physicians to that Institution.

*Diseases affecting particular Organs.*

| | | | | | |
|---|---|---|---|---|---|
| Head and Nervous System | | Apoplexy (decided) | | 2 | |
| | | ——— (threatened) | | 7 | |
| | | Hemiplegia | | 1 | |
| | | Paralysis (of Arm) | | 1 | |
| | | Epilepsy | | 1 | |
| | | Hysteria | | 5 | |
| | | Hypochondriasis | | 2 | |
| | | Chorea | | 1 | |
| | | Tremors | | 2 | =21 |
| Nostrils, Fauces, Mouth, and Throat | | Catarrh | | 17 | |
| | | Cynanche | Tonsillaris | 4 | |
| | | | Laryngea | 2 | |
| | | | Parotidea | 1 | |
| | | Chronic Ulcer of Throat | | 2 | |
| | | Apthæ | | 2 | =23 |
| Organs of Respiration | | Dyspnœa | | 2 | |
| | | Bronchitis | Acute | 4 | |
| | | | Chronic | 51 | |
| | | Pleuritis | | 6 | |
| | | Pneumonia | | 26 | |
| | | Hæmoptisis | | 2 | |
| | | Phthisis | | 12 | =103 |
| Organs of Circulation | | Palpitation (apparently from organic disease) | | 1 | |
| | | Rheumatism of Heart? | | 1 | |
| | | Angina Pectoris | | 1 | = 3 |
| Organs of Digestion | Stomach | Dyspepsia (Simple) | | 9 | |
| | | ——— with Gastrodynia | | 7 | |
| | | ——— with Vomiting | | 2 | |
| | | ——— with Pyrosis | | 2 | =20 |
| | Bowels | Constipation | | 3 | |
| | | Colic | | 1 | |
| | | Diarrhœa | | 4 | |
| | | Dysentery | | 5 | =13 |
| | Liver | Hepatitis | Acute | 1 | |
| | | | Chronic | 3 | |
| | | Icterus | | 3 | = 7 |
| Organs of Urine | | Nephralgia | | | 3 |
| Organs of Generation | | Dysmenorrhœa | | 1 | |
| | | Amenorrhœa | | 2 | |
| | | Leucorrhœa | | 5 | = 8 |
| Skin—Eruptions | Acute | Variola | | 2 | |
| | Chronic | Herpes | | 1 | |
| | | Urticaria | | 2 | |
| | | Psoriasis | | 1 | |
| | | Purpura | | 1 | = 7 |
| Muscles, Tendons, Joints, &c. | | Gout | | 3 | |
| | | Rheumatism | Acute | 11 | |
| | | | Chronic | 16 | =30 |

*Diseases not easily referred to particular Organs.*

| | | | |
|---|---|---|---|
| Fevers | Continued | 10 | |
| | Intermittent | 1 | =11 |
| Dropsies | Ascites | 2 | |
| | Hydrothorax | 1 | |
| | General Dropsy | 3 | = 6 |
| Names of Diseases not registered | | | 12 |
| | Total | | 276 |

Fatal Cases:—Apoplexy, 2; Fever, 1; Bronchitis, 3; Hydrothorax, 1; Phthisis 2; Pneumonia, 1; = 10.

During part of the month of June the thermometer rose to 85°, and in January it sunk to 12°; giving a difference of temperature amounting to 73°!

By referring to the Report of Diseases for the quarter from May to August, 1822, (vol. xlviii. page 267,) it will be perceived that the number of admissions during three months of the warmest part of the season, and three months of the intensely cold weather we have lately experienced, differs but very little. In the former period, the admissions amounted to 301; in the latter, to 276. The very slight variation in the number of patients between one quarterly period and another, shows that there are certain causes constantly in operation, independently of the state of the weather and prevalence of disease, in keeping up the number of patients at public Institutions in London. For five years, the number of admissions at the Westminster General Dispensary has averaged 5000 annually; and the proportion of these falling under my care, as one of the physicians, has scarcely varied more than from 250 to 300 in each month. It is therefore obvious, that it is from the comparative severity or mildness of the diseases, and not from the number of patients, that any judgment can be formed of the state of the public health, and the comparative salubrity of different seasons.* Some interesting information may be obtained by comparing the list of diseases prevalent during the very hot weather of last summer, and the same period of the severe winter which followed. During the first period, the number of patients affected with diseases of the respiratory organs amounted to 41, during the second to 103; of the digestive organs, in the former period 101, in the latter 40. These are by far the most important and striking changes, showing the organs which principally suffer from great variations of temperature. On the other hand, the functions of certain organs seem to have been but little influenced by these atmospheric changes: for example, during the summer quarter, the number of admissions for diseases of the head and nervous system generally amounted to 29, and during the like period in winter, to 22; of the organs of circulation, 4 in the former period, and 3 in the latter; simple continued fevers, 13 and 10; dropsies, 8 and 6; and, what might not perhaps have been expected, the number of rheumatic patients was greater during the summer than the winter quarter, being 37 and 30: there was, however, a larger proportion of acute cases among the latter. The severity of the diseases, it will be observed, corresponds to the

* This I have pointed out more fully in a previous Report, vol. xlvi.

importance of the parts affected; and, accordingly, in the former of the Reports we are comparing, the number of fatal cases amounts to 5, in the latter to 10, of which seven were diseases of the lungs.

The following is the history of the case marked in the table as one of Rheumatism of the Heart:—A man, about forty-five, who had, by his own account, been subject to violent attacks of pain in the left side of the chest, was suddenly seized with very acute pain in the region of the heart, accompanied with intense anxiety and difficulty of breathing, so great as to prevent him from being able to lie down. At the end of six hours, pain began to affect the right shoulder, extending very soon to the elbow; this gradually became more and more severe, the agony about the heart diminishing in proportion. The shoulder next became relieved, the elbow joint remaining extremely painful, and considerably swelled, with very perceptible redness of the skin. It is worthy of remark, that the pain in the region of the heart remained without diminution till the arm became affected; and, by the time the elbow-joint swelled, there remained only a degree of uneasiness in the chest, to which the patient is habitually subject. The attack came on late on Saturday night; the heart became relieved on Sunday morning; the affection of the elbow was at its height on Tuesday, from which time the patient gradually got well. The remedies employed were bleeding and colchicum; but, as the symptoms had become milder before their employment was begun, it is very questionable how far they contributed to the recovery. It has been supposed by some pathologists, that rheumatism of the heart is associated with that form of the disease which attacks muscular parts and fibrous membranes: if this be admitted as a rheumatic affection of the heart, it would tend to disprove this as a remark of universal application. It must also be placed among the more rare and favourable varieties of metastasis,—viz. from the more to the less important organ; the usual change in the seat of inflammation being from external to internal parts, particularly in rheumatism.

Another patient afforded illustration of the extent to which medicines which we are accustomed to exhibit with caution, may sometimes be taken with impunity. A woman, aged fifty, who had been taking thirty drops of the vinum colchici three times a-day, with some benefit, for rheumatism, became impatient of the slowness of her recovery, and, wishing to hasten it, resolved to increase the dose of her medicine. She took two teaspoonsful at nine o'clock in the morning, and one and a half teaspoonful more before two o'clock. I was sent for, and saw her between three and four, when I found her alarmed, but not labouring under any other unpleasant symptom than some nausea and griping pain of the bowels: these, being rather confined, were opened with castor-oil, and next day she was well, so far as regarded the colchicum. The preparation was the wine of the root, and made by Mr. Garden, of Oxford-street.

Another instance of a powerful drug exhibited in an over-dose, did not terminate so favourably. On the evening of the 17th ultimo, I was requested to see a child, which however was dead before my arrival. The history is as follows:—Several children in the same house laboured under small-pox, which had prevailed among them for about

3

a fortnight before the birth of the subject of the present case. The infant, when born, is said to have had some appearance of *pocks;* these, however, were extremely few, and of a very mild kind. On the evening of the eighth day, an officious neighbour gave the child a teaspoonful (which held about one drachm) of syrup of poppies. The infant fell asleep, and, not waking during the night, an intelligent surgeon was sent for, who administered various remedies; but coma had supervened with occasional convulsions, in which state the infant survived till the evening of the second day, being about forty-eight hours from the exhibition of the narcotic. I proceeded, along with the surgeon, to examine the body, about thirty hours after death. The external surface all over the body was darker than natural; the lips and nails were of a deep purple, approaching to black: these appearances had been remarked before the child's death. On the back part of the neck and left shoulder, there were about a dozen pustules, varying in size, the largest equalling that of a split-pea: they had no central depression; neither any hardness nor inflammation surrounded them; the fluid they contained was more like milk than pus. On raising the skull-cap, signs of great turgescence presented themselves; the minute vessels of the arachnoid giving the appearance of the most delicate net-work, of a florid colour. The interstices between the convolutions contained a serous effusion, which oozed out on puncturing the membrane. About two drachms of fluid, of a red colour, was found in the ventricles; probably this appearance arose from the admixture of blood, as the extreme softness of the brain render it difficult to avoid lacerating vessels during the dissection. The air-passages, from the epiglottis downwards, as well as the lungs, were healthy: perhaps these last contained rather more blood than usual. The heart was much distended, particularly the auricles, which were gorged with black blood. The stomach was slightly distended with flatus; its external appearance presented nothing remarkable. On opening it, about a tablespoonful of fluid, exactly resembling pus, was observed smeared over the surface, particularly at the great extremity: when this was wiped off, the internal membrane was found to be red throughout, the colour being of the brightest scarlet at the place where there was the greatest quantity of the purulent covering. The small intestines likewise presented the appearance of inflammation; the large did not. I believe death to have been produced by the medicine, and not by the small-pox,—because the surgeon who attended the child was of that opinion; because the child was immediately thrown into a state of sleep, or stupor, from which it did not subsequently recover; because the small-pox, up to the time of giving the poppies, had never excited any alarm, nor any considerable indisposition: because the appearance of the eruption, described above, indicate a very mild form of the disease; and because the internal examination discovered those parts to be sound, the inflammation of which has been shown to be the most frequent cause of death in cases of small-pox terminating fatally at this period of the disease.

